# Systems Biology for Biologists

**DOI:** 10.1371/journal.ppat.1004786

**Published:** 2015-05-14

**Authors:** Rachel A. Hillmer

**Affiliations:** Department of Plant Biology, Microbial and Plant Genomics Institute, University of Minnesota, Saint Paul, Minnesota, United States of America; Washington University School of Medicine, UNITED STATES

## Have You Been Put Off by Systems Biology?

Do you avoid papers thick with mathematical details and unfamiliar statistical analyses? If so, this article is for you! Systems biology, at its core, is not a set of computational and mathematical techniques; these are merely tools, incredibly useful, but secondary. The heart of systems biology is simple: explaining how a system works requires an integrated outlook. For any phenotype—molecular, macroscopic, or ecological—a set of interrelated factors exist that contribute to this phenotype. Since these factors interact, they need to be studied collectively, not merely individually. That’s it!

## What Is a System?

A system is a collection of parts and factors that work together to complete a task. Conversely, for a given task, the system is defined by the set of all parts and factors which influence, accomplish, or impede that task.

Some systems are easy to identify. Think of a machine, like a car. The body of the car houses all the parts that make up the automobile. The external boundary makes the system easy to identify. Some systems are less easily identified. Consider all the factors that influence traffic flow in a city. The first example is concrete, the second more abstract; both are systems.

## What about Some Biological Systems?

In the systems biology literature, the most commonly discussed systems are networks of genes or proteins. Sometimes these are very large systems: the set of all genes in an organism and the spatiotemporal control of these genes (e.g., [1–3]). But there is no fixed scale at which systems biology operates. Your system could be an ecosystem of plants and the soil services they provide and require; your system could be an epidemiological system with hosts, pathogens, and vectors. Your system could be a single molecular process, like the regulation of an important gene. Or it could be a complex system like the induction of an immune response within a cell, tissue, or organism. If there is a biological question you wish to ask, or a process you wish to study, there is, de facto, a set of parts which contribute to that process; these parts define the system. Biological parts are interconnected and interdependent. Systems biology recognizes this and provides tools and frameworks to both accurately capture these relationships and deduce the system behavior that emerges from these relationships.

## My System Has Tens of Thousands of Molecular Parts. Aren’t Your Claims That Systems Biology Will Help Me Just Wild Speculation?

**Fig 1:**
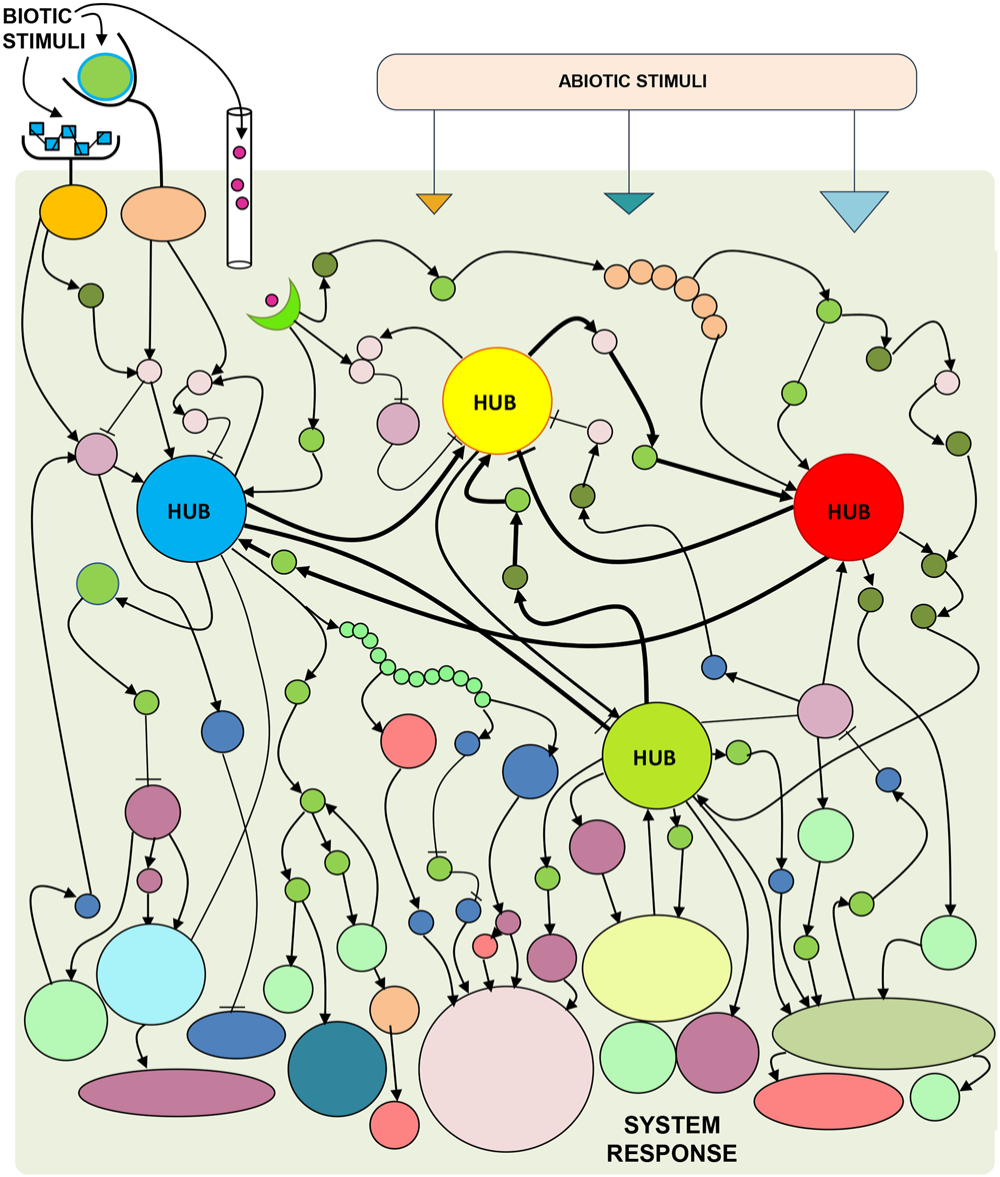
Combinations of system perturbations assist in discovering the mechanisms that drive complex biological system responses. Complex systems, e,g., the immune system of an organism, are notably complicated in two ways: (1) they are tuned to respond differently to different system inputs, and (2) the system that mediates outputs as a function of inputs is full of network redundancy, which ensures operation under nonideal circumstances. Thus, learning the rules for how a complex system operates requires coincident, varied, and likely combinatorial external and internal perturbations to a system. Mild perturbations are likely buffered by the system; strong perturbations are the key. Complex systems often have numerous parts; how do we decide which parts should be perturbed? Network hubs—parts which integrate numerous signals and/or regulate many parts—are excellent candidates for an abbreviated parts list. We then monitor system behavior, in response to external perturbations, when different combinations of hubs have been rendered inoperable. Data from this monitoring can enable mathematical reconstruction of how major system parts are stimulated, influence each other, and modulate system output. These mathematical models are then used to direct further experiments, which leads to model refinement. Goals of iterative model building include: furthering our fundamental understanding of the system and predicting properties of the system relevant in applied settings. Image: conceptual diagram of a complex system that responds to system inputs (i.e., external perturbations).

Happily, no. The solution: taking stock of major effects and ignoring minor ones. Good systems biology is a balance between reductionism—breaking a system apart into smaller parts and defining the function of these parts—and synthesis—understanding how the parts cooperate to produce the behavior of the whole. We have two options: (1) discover and study small modular subsystems [4] or (2) approximate a complex system via a tractable number of components [5] (e.g., [6]). To do the latter, we first look for the parts which have large effects on our process or phenotype of interest, so-called “large-effect” parts [7]. For example, if the system is an organism-level process, these may be hormone concentrations, which by definition regulate a large number of molecular processes, e.g., [6,8]. Or, they may be cells in the circulatory system, which can monitor and regulate multiple tissues, e.g., [9]. The hubs of a complex system serve as excellent candidates for major-effect parts (Fig 1). Hubs, by definition, do either or both of the following: (1) integrate regulatory information from many parts or system inputs, (2) transduce this information to regulate multiple processes or parts. If we first get a good approximation of the basic functionality of a system, we can then add on the bells and whistles.

To figure out the major-effect parts and/or processes of your system, there are a host of established biological methods, including:

1. observation
2. forward genetic screens
3. genome-sequence-assisted guesses
4. gene expression analysis
5. an external system perturbation (e.g., exogenous chemical application)

## How Do I Define the Rules Governing My Biological System?

Once you have a first-pass parts list assembled, you will need to combine two types of experimental factors:

1. external system perturbations
2. internal system perturbations

Why are perturbations needed? As in classical genetics, we learn about systems best by breaking or aggravating them in defined ways, observing how those induced changes modulate the process or phenotype of interest. External perturbations include, for example, treating a tissue with pathogens or pathogen-derived compounds. An internal perturbation involves removing, disabling, or modifying one or more system parts. Diverse internal system perturbations are needed because complex, robust systems are often full of redundancies and backups. Robust systems buffer mild perturbations. Ideally, a combinatorial set of internal perturbations that jointly abolish a phenotype would be challenged by a representative diverse suite of external system perturbations that stimulate the system in different precise ways [10] (e.g., [6,11]).

The system should be measured across appropriate timecourses to capture when the system is dynamically responding to the (especially external) perturbations. Quantitative monitoring of both the system parts and the system output is ideal. Such data empowers mathematical deduction of the mechanisms by which system parts control and modulate the system response.

## What Is a Mathematical Model?

A mathematical model is a set of relationships, usually written as equations, that describe how the parts of the system respond to system inputs, regulate each other, and control system output. Why do we need math to do this? Math is just formalized logic, so in theory we could just use descriptive sentences. But for all except the simplest relationships, exhaustively working out the implications of these relationships is prohibitively laborious and error-prone. Moreover, language can be imprecise, where math naturally tends towards precise expression of relationships. Why is a model valuable? Models are hypothesis generation tools, efficient ways to scout out novel and interesting system behavior. We use them to explore in silico varied external conditions and internal system modifications. Accomplishing such exploration experimentally is usually far more labor intensive, costly, and perhaps even impossible. A good mathematical model is an imperfect but useful virtual copy of a system that reproduces the salient features of the system. This copy lets us play with the system using computational techniques, analogous to how physical toy models help chemists think about the structure of a molecule.

Perfect system knowledge is not a prerequisite for starting to build a mathematical model. Model building is an iterative procedure: model, predict, test experimentally, repeat all. Modeling, when done well, will help channel further experiments in the most fruitful directions.

Have no fear, you do not need to become an expert in math. You need only make friends with someone who is. And your collaborator very much needs your input on the model. Mathematical approaches and structures need to be chosen which capture and reflect the essence of each biological system. For this, the training and intuition of a biologist is irreplaceable.

## Are There Any Systems Biology Success Stories?

Why, yes, indeed there are. Successful mathematical modeling of biology has a long history that began long before the genomics era. Here are some highlights. Tissue models of the human heart stand on over half a century of iterative modeling, experimentation, and model refinement. Birthed from this long labor, the virtual heart, used in clinical settings, may be systems biology’s brightest star [12,13]. In 1952, British mathematician Alan Turing proposed that leopard spots, zebra stripes, and spirals in nature could arise by a simple reaction-diffusion equation imposed on a homogenous system [14]. It took decades to develop the molecular tools to test his hypothesis, but he was right [15,16]. During the 2001 United Kingdom outbreak of foot-and-mouth disease, mathematical models were used to predict disease spread and assisted in deciding control measures [17]. An integrated biomedical informatics program, aneurIST, predicts rupture of incidentally discovered cerebral aneurysms using additional patient-specific medical data. During active model development, it was estimated that this modeling effort saved thousands to millions of euros annually in unnecessary procedures [18]. A few excellent molecular systems biology models are described on Nicolas Le Novère’s blog [19]. For numerous additional examples, see the European Bioinformatics Institute’s (EMBL-EBI) “Models of the Month” database, part of its BioModels database [20].

## Should You Become a Systems Biologist?

Are you perfectly content to study a small system? There’s no pressure to take on a wildly ambitious system, understanding the function of an entire cell, or modeling the ecosystem of planet Earth. For any biological question, a relevant system exists; the study of this system will benefit from including mathematical models in your toolkit.

You may be hesitant to consider becoming a systems biologist. Math fear is a real thing. But who knows? There might be a collaborator waiting for you just across campus. From my experience as a physicist turned biologist, I can confidently say there are mathematicians, physical and computer scientists, and engineers who have been lured by the extraordinariness of biology.

**Further Reading**

1. Wolkenhauer O. Why model? Front Physiol. 2014 Jan 28; 5: 21.
2. Wolkenhauer O, Fell D, De Meyts P, Blüthgen N, Herzel H, Le Novère N et al. SysBioMed report: advancing systems biology for medical applications. IET Syst Biol. 2009 May; 3(3): 131–6.
3. Cohen JE. Mathematics is biology’s next microscope, only better; biology is mathematics’ next physics, only better. PLoS Biol. 2004 Dec; 2(12): e439.
4. Kitano H. Computational systems biology. Nature. 2002 Nov 14; 420(6912): 206–10.
5. Wingreen N, Botstein D. Back to the future: education for systems-level biologists. Nat Rev Mol Cell Biol. 2006 Nov; 7(11): 829–32.
